# Pragmatic open-label clinical trial investigating the treatment effects of Ginkgo biloba extract EGb 761 on patients with vertigo and dizziness of various aetiologies

**DOI:** 10.3389/fphar.2026.1824426

**Published:** 2026-07-24

**Authors:** Grażyna Lisowska, Irena Urban, Sandra Schlaefke, Andreas Zwergal, Piotr H. Skarżyński

**Affiliations:** 1 NZOZ Centrum Medyczne LIMED, Tarnowskie Góry, Poland; 2 Centre of Hearing and Speech MEDINCUS, Kajetany, Poland; 3 Clinical Research, Dr. Willmar Schwabe GmbH & Co. KG, Karlsruhe, Germany; 4 Department of Neurology, LMU University Hospital, LMU Medizin, LMU Munich, Munich, Germany; 5 German Center for Vertigo and Balance Disorders (DSGZ), LMU University Hospital, LMU Medizin, LMU Munich, Munich, Germany; 6 Institute of Sensory Organs, Kajetany, Poland

**Keywords:** dizziness, effect modifiers, effectiveness, ginkgo biloba extract EGb 761, pragmatic clinical study, vertigo

## Abstract

**Introduction:**

Ginkgo extract EGb 761 demonstrated favourable therapeutic effects in peripheral and central vestibular syndromes. This pragmatic study explored whether administration of EGb 761 in patients with vertigo and dizziness of various aetiologies results in improvement of symptom intensity, posture, gait and ocular motor function, and whether specific patient subgroups benefit most from this treatment.

**Methods:**

This pragmatic open-label, multicentre clinical trial involved 179 patients with various vertigo and dizziness syndromes taking 240 mg of EGb 761 daily for 12 weeks. Patient-reported outcomes were assessed at 6 and 12 weeks using the Dizziness Handicap Inventory (DHI), the short form of the Vertigo Symptom Scale (VSS-SF) and a 11-point box scale to measure vertigo severity. A moderate treatment response was defined as an improvement of at least 30% from the baseline score. The Romberg test and Unterberger stepping test were carried out to clinically grade posture and gait instability. Spontaneous nystagmus detection was performed with and without fixation suppression. Subgroups were analysed using analysis of covariance models to explore potential effect modifiers.

**Results:**

A total of 174 patients (mean age: 52.2 years; 77.6% female) were enrolled. Compared to baseline, mean scores on the DHI, the VSS-SF and the 11-point box scale for vertigo severity showed statistically significant improvement (p < 0.0001) both after 6 and 12 weeks. After 12 weeks, two-thirds of patients (between 60.9% and 66.7%) showed a clinically relevant improvement in these patient-reported outcomes. The proportion of patients with a normal postural control in the Romberg test increased from 48.3% at baseline to 85.2% after 12 weeks. Meanwhile, the proportion of patients with abnormal results in the Unterberger stepping test decreased from 33.5% at baseline to 10.1% at week 12. Patients with episodic vertigo/dizziness, and those whose symptoms had lasted up to 6 months, improved more than those with continuous vertigo/dizziness and symptoms lasting more than 6 months.

**Conclusion:**

After 12 weeks, patient-reported and functional outcomes had significantly improved in patients with vertigo/dizziness. However, within the setting of this pragmatic study, it is not possible to discriminate between the effects of treatment, self-healing, or the natural course of the disease.

**Clinical Trial Registration:**

https://eudract.ema.europa.eu/, identifier EUdraCT 2016-000316-15.

## 1 Introduction

Vertigo and dizziness are very common health issues that are frequently reported in primary care, with a higher incidence among female patients than male ones. A study of a nationally representative sample of 4,869 German adults aged 18–79 found that the 1-year prevalence of moderate to severe dizziness was 22.9%. The lifetime prevalence of dizziness-related medical consultations was 17.1% (Neuhauser, 2016). A large population-based cohort study (the Gutenberg Health Study at University Hospital Mainz in Germany) involving 8,519 participants found that 21.6% of participants had experienced vertigo (Hackenberg et al., 2023). The prevalence of vertigo and dizziness increases significantly with age, affecting approximately 30% of individuals over 60 years of age and up to 50% of those over 85 (Fancello et al., 2023).

The term ‘vertigo’ refers to the sensation of movement in the subject or their surroundings without any actual movement, while ‘dizziness’ refers to the sensation of disturbed spatial orientation without any impairment of the sense of motion (Bisdorff et al., 2009). Patients often find it difficult to differentiate between the two symptoms. Consequently, both terms are frequently used together, and current strategies for classifying symptom characteristics focus primarily on the duration of symptoms (i.e., acute, episodic or chronic) (Newman-Toker and Edlow, 2015). It is important to note that vertigo and dizziness are symptoms of various heterogeneous disorders with different aetiologies, ranging from peripheral vestibular and non-vestibular disorders to central nervous system dysfunction. Peripheral vestibular forms are typically associated with benign paroxysmal positional vertigo (BPPV), Ménière’s disease or vestibular neuritis. In contrast, central forms are often associated with vestibular migraine, cerebrovascular events or cerebellar disorders (Brandt and Dieterich, 2017; Feil et al., 2019). In a tertiary dizziness unit, the most common forms of vertigo and dizziness are BPPV (17.1%), functional dizziness (15%), central vestibular syndromes (12.3%), vestibular migraine (11.4%), Menière’s disease (10.1%), vestibular neuritis (8.3%), bilateral vestibulopathy (7.1%), and vestibular paroxysmia (3.7%) (Strupp et al., 2013). In recent years, vertigo has also been reported as a symptom of SARS-CoV-2 infection (Fancello et al., 2021). The virus may directly damage the cochlea and the vestibular labyrinth. Indirect viral damage may result from inflammation in the cochlea and the labyrinth triggered by a persistent pro-inflammatory cytokine response or an autoimmune mechanism (Karimi-Galougahi et al., 2020).

The management of vertigo and dizziness depends on their aetiology and causative diagnosis. However, this cannot be established for a significant proportion of patients in primary and secondary care (Zwergal et al., 2023a; b). The complexity of the aetiological diagnosis stems from the involvement of numerous peripheral sensory organ systems and central nervous functional units, as well as the absence of definitive biomarkers for specific vestibular syndromes. Furthermore, high-level evidence for therapeutic strategies tailored to specific conditions is often lacking (Tarnutzer et al., 2025). Practical treatment concepts for patients with vertigo or dizziness therefore frequently combine balance training with pharmacological interventions and psychological counselling adapted to the needs of the individual patient. Several drugs are used for the symptomatic treatment of vertigo and dizziness, including Ginkgo biloba extracts such as EGb 761 (Hamann, 2007; Tarnutzer et al., 2025).

EGb 761 is a dry extract from Ginkgo biloba leaves with a drug to extract ratio of 35–67:1 manufactured by Dr. Willmar Schwabe GmbH and Co. KG, Germany. The extract is adjusted to 22%–27% Ginkgo flavonoids calculated as Ginkgo flavonol glycosides, and 5.4%–6.6% terpene lactones consisting of 2.8%–3.4% ginkgolides A, B, C and 2.6%–3.2% bilobalide, while it contains less than 5 ppm ginkgolic acids. In earlier randomized, controlled trials, EGb 761 has been found to improve symptoms of patients with peripheral and central aetiologies of vertigo/dizziness (Hamann, 1985; Sokolova et al., 2014; Heide et al., 2022). A systematic review of randomized, placebo-controlled studies concludes that EGb 761 is effective in the treatment of vestibular and non-vestibular vertigo/dizziness (Hamann, 2007). Supportive data on its effectiveness for the treatment of dizziness in patients with cognitive impairment are delivered by a meta-analysis across five large controlled trials (Spiegel et al., 2018). Two additional independent meta-analyses on the efficacy and safety of EGb 761 in cognitive impairment and dementia revealed that the cumulative OR (95% CI) for dizziness as an accompanying symptom was significantly lower in the EGb 761 group than in the placebo group (Hashiguchi et al., 2015; Tan et al., 2015).

Despite these lines of evidence, little is known about predictors of response to symptomatic EGb 761 treatment in vertigo/dizziness syndromes of various aetiology and duration or effect sizes when modern patient-related rating scales and clinical outcome measures are used. Therefore, the present pragmatic study was designed to generate descriptive data that would help to design future randomised controlled trials with EGb 761 in vertigo/dizziness syndromes.

## 2 Materials and methods

### 2.1 Study design and population

The objective of this interventional, uncontrolled, open-label multicentre clinical trial of EGb 761 in the treatment of patients with vertigo or dizziness syndromes of various aetiologies, was 1) to explore whether the dynamics and chronicity of symptoms influence the therapeutic response rates to EGb 761 and 2) to identify groups of patients that benefit most from the treatment. Adult patients suffering from vertigo or dizziness of vestibular origin for a minimum of 2 weeks and had a score of at least 25 in the DHI were enrolled at 12 different sites (otolaryngologists or internists) in Poland.

To confirm vestibular dysfunction (Strupp et al., 2017), standard diagnostic tests such as videonystagmography, including bithermal caloric testing, and video head impulse testing (vHIT) were performed at the screening visit or within 3 months of the baseline visit. Slow phase velocity (SPV) in the caloric test at 6°/second or less was considered an indication of vestibular hypofunction on the side tested. Unilateral vestibular pathology was defined by applying the Jongkees formula with a threshold of 25% as suggested by consensus criteria (Strupp et al., 2022). A vestibulo-ocular reflex (VOR) gain in the vHIT of <0.6 on both sides was used as diagnostic criterion for bilateral vestibulopathy (Strupp et al., 2017). A VOR gain of <0.7 and a difference of >0.3 compared to the healthy side were used as thresholds for unilateral vestibulopathy (Strupp et al., 2022).

Patients with a BPPV were identified by positioning manoeuvres (i.e., the Dix-Hallpike test) and not included in the study. Concomitant guideline-based standard care, such as physical therapy or balance exercises, was permitted.

Patients were instructed to take two film-coated tablets containing 120 mg EGb 761 per day. The treatment period was 12 weeks with face-to-face visits (at weeks 6 and 12).

The study was approved by the Ethics Committee at Silesian Medical Chamber in Katowice and conducted in accordance with the principles of Good Clinical Practice and the Declaration of Helsinki. Written informed consent was obtained from all participants. The study was registered in the database for interventional clinical trials on medicinal products in the European Union (EudraCT 2016-000316-15), which was used until 30 January 2025. The publication manuscript was written in accordance with the STROBE checklist (Strengthening the Reporting of Observational studies in Epidemiology) (von Elm et al., 2007).

### 2.2 Outcomes

The severity of vertigo/dizziness-associated symptoms and the general impairment of daily life were assessed at baseline visit and after 6 and 12 weeks using the DHI, the short form of the Vertigo Symptom Scale (VSS-SF), a 11-point box scale for vertigo severity and the Sheehan Disability Scale (SDS). The DHI has total scores ranging from 0 to 100 with higher scores indicating more severe symptoms (Jacobson and Newman, 1990). It is suitable as quantitative measure for monitoring ongoing treatment and rehabilitation. The DHI is a fully functional questionnaire that meets all the validation criteria for use on Polish patients with vertigo or dizziness (Zamyslowska-Szmytke et al., 2021).

The total score of the VSS-SF ranges from 0-60 with higher scores indicating more severe problems (Yardley et al., 1992). Furthermore, vertigo severity was assessed by patients on an 11-point box scale anchored by word description at each end (0 = no vertigo, 10 = extremely severe vertigo).

The SDS was also employed in this study (Sheehan et al., 1996). It is a self-report scale that evaluates functional impairment in relation to professional, domestic, and familial responsibilities. The assessment uses a 10-point numerical scale, enabling quantitative analysis of performance in these domains.

In addition, three specific clinical examinations for assessment of ocular motor, postural and locomotor signs associated with vertigo or dizziness were carried out. A spontaneous nystagmus test using devices for visual fixation suppression was conducted and rated dichotomously (present/absent). Spontaneous nystagmus was taken as an indicator for asymmetric vestibular dysfunction (Liu et al., 2022). Postural control was assessed by the Romberg test, which is suitable to screen for cerebellar or sensory (i.e., vestibular, proprioceptive) balance disorders. Patient were standing with feet together, arms folded, eyes open and closed. The observer watched for swaying or movement of the feet to attain balance. Patients with vestibular disorders typically show a deterioration of balances in the eyes-closed relative to eyes-open condition (Lanska, 2002). The test was rated negative if there was no undirected swaying and no directed tendency to fall. The Unterberger stepping test was used for a clinical examination of the patient’s standing and gait pattern (Reiss and Reiss, 1997). The patient was asked to step on the spot 50-times with eyes closed and arms outstretched. During the test the investigator observed whether the patient was rotating on his own axis. A rotation of more than 45° was taken as an indicator for an ipsilateral peripheral- or central-vestibular impairment.

The safety and tolerability outcomes encompassed the frequency and nature of adverse events (AEs) throughout the study. The physical examination, safety laboratory tests (haematology, clinical chemistry including liver function and blood coagulation parameters), and vital signs were documented at screening and week 12 visit.

### 2.3 Statistical analysis

In view of the absence of clarity regarding the factors that influence the treatment effect of EGb 761, no formal hypothesis was formulated. Consequently, the data were analysed descriptively.

Baseline characteristics and safety outcomes are presented by absolute frequencies and percentages for categorical variables and by means with standard deviations for continuous variables. The main variables describing treatment effects were the differences in mean scores from baseline to week six visit and week 12 visit for DHI, VSS-SF and vertigo severity (11-point box scale).

Analysis of covariance models were applied using the value observed at each follow up visit as the dependent variable, while the baseline variable was utilized as the covariate. The SDS total score was analysed in the same way. Continuous outcomes were described by the number of evaluable observations (N), arithmetic mean (mean) and standard deviation (SD). P-values of the ANCOVA models are presented.

Any missing values at week 12 were replaced by the last observation carried forward (LOCF) method. For variables composed of single-item variables, the LOCF was applied on an item-by-item basis. Missing values at week six were not replaced by baseline values.

The treatment effects were examined in subgroups defined by baseline characteristics (episodic versus continuous vertigo/dizziness, duration ≤6 months versus >6 months) and analysed using models of covariance as described above with the additional factor “subgroup”. The differences of treatment effects between subgroups are presented by adjusted means, standard errors of adjusted means, and p-values for changes within subgroups, as well as adjusted means differences, standard errors of adjusted mean differences, and p-values for the comparison of subgroups.

For the responder analysis, an improvement between baseline and week 12 by at least 15% of the baseline score was defined as a ‘slight’ response for each of the main outcomes (DHI, VSS-SF, vertigo severity), while an improvement by at least 30% was defined as a clinically relevant response. Responder rates are presented as percentages based on the sample size of the full analysis set.

## 3 Results

**FIGURE 1:**
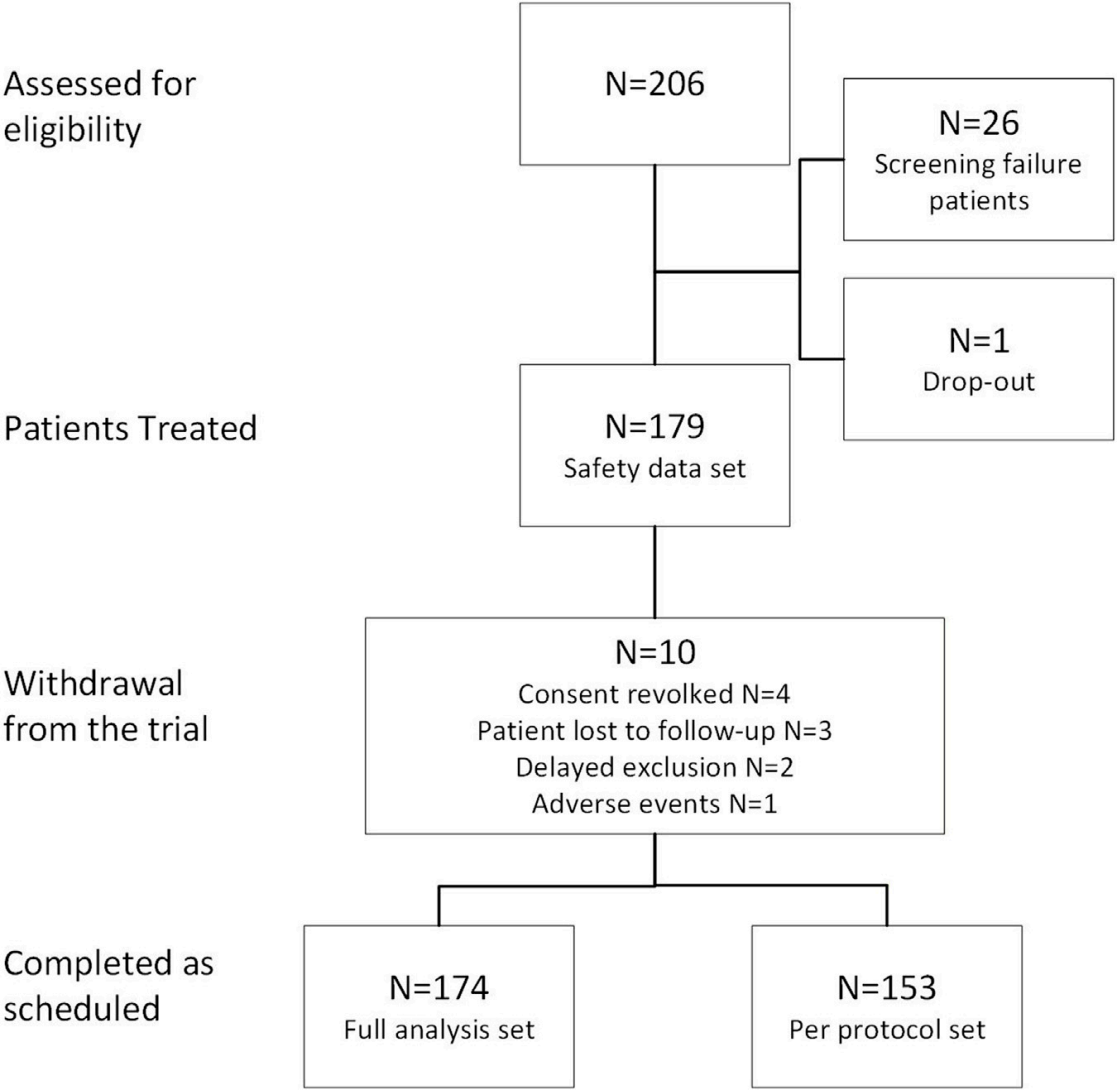
Disposition of subjects, analysis data sets.

In total, 206 patients were screened for enrolment into the trial (Figure 1). Twenty-six patients did not successfully complete the screening phase (screening failure patients). The remaining 180 patients were eligible for trial participation and started - apart from one subject - the intake of EGb 761. The first patient was screened in October 2016 and the last patient completed the trial in February 2018. All 179 patients who started treatment with EGb 761 were included in the statistical analysis of safety. Five of these patients had no post-baseline values for the main treatment effect variables and were therefore excluded from the full analysis set (FAS).

Data from 174 patients (FAS) were analysed with respect to efficacy. The baseline characteristics of these patients are presented in Table 1. Of the 173 patients in the FAS who underwent the caloric test, 107 had no abnormal findings, 59 showed an indication of unilateral vestibulopathy, and seven showed an indication of bilateral vestibulopathy (see Table 1).

**TABLE 1:** Patient baseline characteristics.

Parameter	Total (N = 174)
Age (years) Mean ± SD Min, max	52.2 ± 14.5 20, 85
Gender, n (%) Female Male	135 (77.6%) 39 (22.4%)
Risk factors, n (%) Patients with cardiovascular disease Patients with vascular risk factors	19 (10.9%) 71 (40.8%)
Vertigo/dizziness duration and time course, n (%) ≤6 months >6 months Episodic Continuous	31 (17.8%) 143 (82.2%) 146 (85.6%) 25 (14.4%)
Clinical diagnosis, n (%) Peripheral-vestibular Central-vestibular	140 (80.5%) 34 (19.5%)
Bithermal caloric testing, n	​
Normal findings	107
Unilateral vestibular hypofunction	59
Bilateral vestibular hypofunction Missing values	7 1
vHIT overall assessment, n	​
Normal findings	143
Unilateral vestibular hypofunction	16 (left), 12 (right)
Bilateral vestibular hypofunction	3

The mean (±SD) VOR gain for the FAS (n = 174) was 0.9 ± 0.27 (range: 0–2) for the left ear and 1.0 ± 0.28 (range: 0–2) for the right ear. Catch-up saccades were present in 24 patients (13.8%). The overall assessment based on vHIT indicated a unilateral vestibulopathy in 28 patients and a bilateral vestibulopathy in three patients (Table 1).

### 3.1 Changes of patient-reported outcomes

Valid data on DHI and vertigo severity were available for all 174 patients and on VSS-SF for 170 patients at baseline, week six and week 12 visit.

**FIGURE 2:**
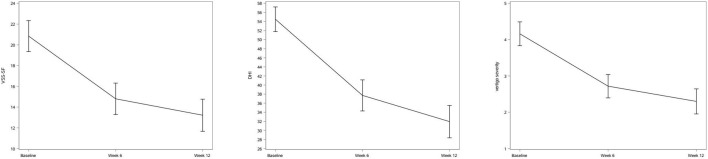
DHI total score, VSS-SF and vertigo severity (11-point box scale) during the trial (mean and 95% confidence interval) ion the FAS (N = 174).

The baseline scores (mean ± SD) were 20.8 ± 9.9 points for VSS-SF, 54.5 ± 18.1 points for DHI, and 4.2 ± 2.2 points for vertigo severity. Compared to baseline, mean scores of VSS-SF, DHI and vertigo severity showed a statistically significant improvement (p < 0.0001) both after 6 and 12 weeks in the ANCOVA model with baseline value as covariate (Table 2; Figure 2). The mean SDS total score (±SD) also decreased statistically significantly from baseline 11.5 ± 7.5 score points to 6.1 ± 6.6 score points at the week 12 visit (p < 0.0001).

**TABLE 2:** Overall treatment effects (FAS): VSS-SF, DHI, vertigo severity (11-point box scale).

Outcome	Baseline		Difference week 6 visit - baseline			Difference week 12 visit - baseline		
	Mean	± SD	Mean	± SD	p-value	Mean	± SD	p-value
VSS-SF (N = 171)^1^	20.8	±9.9	−6.1	±8.3	<0.0001	−7.8	±9.5	<0.0001
DHI (N = 174)	54.5	±18.1	−16.8	±18.0	<0.0001	−22.6	±19.7	<0.0001
Vertigo severity (N = 174)	4.2	±2.2	−1.4	±2.1	<0.0001	−1.9	±2.4	<0.0001

VSS-SF, Vertigo Symptom Scale - Short Form; DHI, Dizziness Handicap Inventory.

1 In week six and week 12 data of VSS-SF, were available for N = 170 patients.

### 3.2 Influence of symptom trajectories on patient-reported outcome

A statistically significant difference (p < 0.05) between the episodic and continuous vertigo/dizziness subgroups was obtained in the ANCOVA at the week 12 visit regarding the two main variables: VSS-SF (p = 0.0053) and DHI (p = 0.0027). A trend for a meaningful difference between the subgroups was observed for vertigo severity (p = 0.1016). The results indicate a superior improvement in the subset of patients with episodic compared to the subset of patients with continuous vertigo/dizziness.

For the factor duration of vertigo/dizziness, a statistically significant difference (p < 0.05) between the adjusted means of the two subgroups (≤6 months vs. >6 months) was obtained in the ANCOVA model regarding the DHI at the week 12 visit (p = 0.01998). The mean reduction in DHI scores indicates a reduced disability in the subset of patients with symptoms lasting up to 6 months, in comparison to the subset of patients with symptoms that persisted beyond 6 months.

### 3.3 Changes of clinical examination outcomes

The Romberg test was performed on 174 patients at their initial screening visit and on 169 patients at their week 12 visit. The proportion of patients showing no swaying increased from 48.3% at baseline to 85.2% after 12 weeks. During this period, the proportion of patients, who tended to fall decreased from 2.3% to 0.0%.

The Unterberger stepping test was performed on 173 patients at the screening visit and on 169 patients at the week 12 visit. The proportion of patients with positive results (i.e., rotation of more than 45°) decreased from 33.5% at baseline to 10.1% at week 12.

The spontaneous nystagmus test was performed on 174 patients at the screening visit and on 170 patients at the week 12 visit. The proportion of positive results without visual fixation suppression by Frenzel glasses decreased from 1.1% at baseline to 0.6% at week 12. Similarly, the frequency of positive results with fixation suppression decreased from 6.9% at baseline to 1.7% at week 12 (Table 3).

**TABLE 3:** Overall treatment effects in FAS on clinical examination outcomes.

Test procedure	Baseline	12 weeks visit
Romberg test No swaying Tendency to fall	48.3% (84/174 patients) 2.3% (4/174 patients)	85.2% (144/169 patients) 0.0% (0/169 patients)
Unterberger stepping test Positive (rotation more than 45°) Negative (no rotation or at least 45°) Missing information	33.5% (58/173 patients) 66.1% (115/173 patients) 0.6% (1/173 patients)	10.1% (17/169 patients) 89.3% (151/169 patients) 0.6% (1/169 patients)
Nystagmus test with frenzel glasses (or videonystagmography) Positive result (abnormal) Negative result (normal)	6.9% (12/174 patients) 93.1% (162/174 patients)	1.7% (3/170 patients) 98.3% (167/170 patients)
Nystagmus test without frenzel glasses Positive result (abnormal) Negative result (normal)	1.1% (2/174 patients) 98.9% (172/174 patients)	0.6% (1/170 patients) 99.4% (169/170 patients)

### 3.4 Responder analysis

**FIGURE 3:**
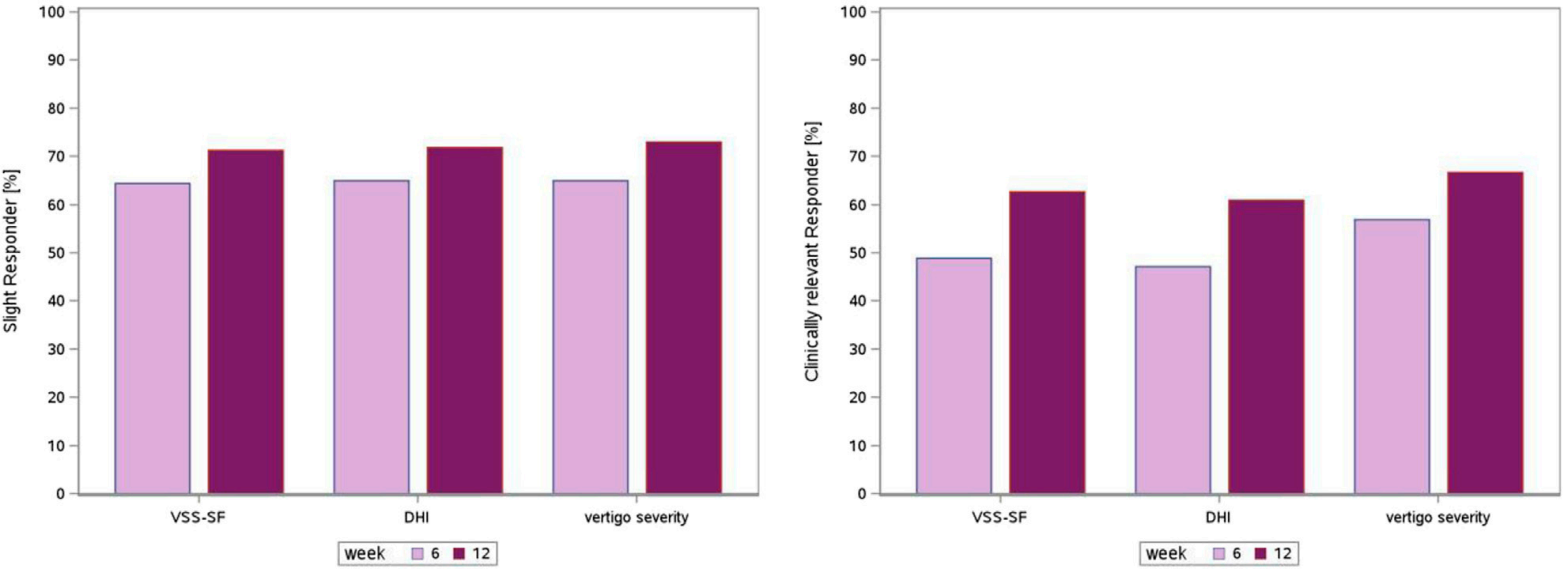
Responder analysis–percentage of patients with a **(A)** slight or a **(B)** clinically relevant response after 6 and 12 weeks (N = 174).

Response rates (≥15% improvement over the VSS-SF, DHI and vertigo severity baseline score) were between 64.4% and 64.9% at the week six visit and increased to 71.3%–73% at the week 12 visit (Table 4; Figure 3). Approximately half of the patients were responders with ≥30% improvement at the week six visit (range: 47.1%–56.9%) and approximately two-thirds of patients were responders at the week 12 visit (range: 60.9%–66.7%). The mean percentage score improvements were higher for DHI and vertigo severity compared to VSS-SF both at the week six visit (32.0% and 28.4% vs. 26.9%, respectively) and the week 12 visit (43.0% and 43.5% vs. 34.8%, respectively).

**TABLE 4:** Responder analysis (FAS, N = 174).

Variable	Category	Week 6 visit N (%)	Week 12 visit N (%)
VSS-SF	≥15%	112 (64.4)	124 (71.3)
	≥30%	85 (48.9)	109 (62.6)
	Mean improvement (%)	26.9	34.8
DHI	≥15%	113 (64.9)	125 (71.8)
	≥30%	82 (47.1)	106 (60.9)
	Mean improvement (%)	32.0	43.0
Vertigo severity	≥15%	113 (64.9)	127 (73.0)
	≥30%	99 (56.9)	116 (66.7)
	Mean improvement (%)	28.4	43.5

VSS-SF, Vertigo Symptom Scale - Short Form; DHI, dizziness handicap inventory.

Subgroup analyses showed that improvement during treatment with EGb 761 was better in patients with episodic vs. continuous vertigo/dizziness, and patients with symptoms ≤6 months vs. >6 months.

### 3.5 Safety analysis

The treatment with EGb 761 for 12 weeks was safe and well tolerated. The mean treatment compliance of the 170 patients with valid data was 97.6% ± 7.8%. The mean duration of exposure to the study medication was 83.4 ± 14.7 days (safety analysis set, n = 178 patients with evaluable data).

During treatment and the following 2 days after the last dose of EGb 761, 34 patients (19.0%) of the safety set (n = 179) experienced a total of 62 AEs with mostly mild or moderate intensity. The most frequent AEs independent from causality were infections and infestations (n = 16 patients) followed by nervous system disorders (n = 9 patients) and gastrointestinal disorders (n = 8 patients). Two patients experienced two serious AEs, which were considered as unrelated to treatment with EGb 761.

For 25 AEs affecting 18 patients, a causal relationship with the study drug could not be excluded. These were most frequently nervous system disorders (n = 6 patients) and gastrointestinal disorders (n = 6 patients) followed by ear and labyrinth disorders (n = 2 patients), general disorders (n = 2 patients), administration site conditions (n = 2), respiratory, thoracic and mediastinal disorders (n = 2 patients). All suspected adverse drug reactions were mild or moderate in intensity and resolved by the end of the study or during follow-up. The causal relationship for the vast majority was assessed as unlikely.

No clinically relevant changes in vital signs or by physical and Ear-Nose-Throat examination were observed in any patient. No relevant changes of mean, minimum and maximum for any safety laboratory parameter (haematology, clinical chemistry including liver function, blood coagulation and urinalysis) from the screening to the week 12 visit were seen.

## 4 Discussion

This multicentre, uncontrolled, open-label, exploratory clinical trial involved a large cohort of patients with various vertigo and dizziness syndromes taking 120 mg of EGb 761 twice daily for 12 weeks. The main finding was that the patient-reported outcome variables VSS-SF, DHI, and vertigo severity significantly had improved after 12 weeks. Almost three-quarters of patients showed a slight response, and approximately two-thirds showed a clinically relevant therapeutic response. Consistently, clinical signs of ocular motor, postural, and gait control improved during the study. A superior improvement was observed in the subset of patients with episodic vertigo/dizziness compared to chronic vertigo/dizziness, and in patients with a symptom duration of less than 6 months. The results of this open-label study are comparable to those of a controlled, double-dummy study of the same duration. Patients presenting with vertigo of various aetiologies were randomised to receive either EGb 761 or betahistine alongside matched placebos for 12 weeks. The authors concluded that both treatments were equally effective, meaning that EGb 761 could be used as an alternative to betahistine (Jianbunjongkit et al., 2026). The baseline DHI score in the betahistine group (54.3 ± 16.9) was like that in our study (54.5 ± 18.1), whereas in the EGb group (63.7 ± 17), it was significantly higher.

The study demonstrated that EGb 761 intake was safe and well tolerated, and that patients adhered to the treatment well. This finding is consistent with an updated umbrella review on the safety of ginkgo extract. Analysis of adverse events showed they were mild with no significant differences compared to placebo groups (Fan et al., 2022). In very rare cases, EGb 761 may cause mild gastrointestinal disturbances, hypersensitivity reactions or headaches. Bleeding from single organs has been documented in postmarketing surveillance. Patients with blood coagulation disorders, those taking anticoagulant drugs and patients with epilepsy should exercise caution when taking this medication.

The present study enrolled a diverse group of patients presenting with various vertigo and dizziness syndromes. Most patients experienced episodic symptoms of peripheral vestibular origin and had had the condition for more than 6 months, suggesting chronic vertigo/dizziness syndromes. Patient-reported symptom burden was moderate at baseline. Female patients were overrepresented. Thus, the composition of the study cohort resembled the characteristics of patients commonly seen in specialised outpatient care settings for patients with dizziness (Brandt and Dieterich, 2017; Zwergal et al., 2023a; Xing et al., 2024), suggesting that it was representative of a secondary-to-tertiary care context.

It is worth noting that the study cohort was recruited using a symptom-based rather than a disorder-based inclusion approach. While this results in some heterogeneity of the underlying pathophysiology, the common denominator was suspected vestibular origins of the symptoms, substantiated by baseline quantitative vestibular function testing. Thus, the treatment approach was symptomatic rather than causative. Previous studies have shown that approximately half of patients presenting with episodic or chronic vertigo/dizziness will remain undiagnosed in primary care (Zwergal et al., 2023b), necessitating a symptom-centred treatment strategy.

The high proportion of patients who responded well to the treatment may be explained by the polyvalent pharmacodynamic effects of EGb 761 on vestibular and equilibrium-related neural circuits in both the central nervous system and the inner ear. Nonclinical investigations in cell cultures and animal models have highlighted the therapeutic potential of EGb 761 to increase neural plasticity and enhance neuroprotection by propagating synaptic connectivity, attenuating age-related deficits in neurotransmitters, improving microcirculation and blood rheology, reducing neuroinflammation, scavenging free radicals, enhancing mitochondrial function and promoting neurogenesis (Muller et al., 2012; Morató et al., 2024).

Specifically, EGb 761 has been shown to speed up vestibular compensation following unilateral labyrinthectomy or vestibular neurectomy in animal studies (Tighilet and Lacour, 1995; Lindner et al., 2019). EGb 761 enhances the recovery of spontaneous nystagmus, postural asymmetry and locomotor deficits by modulating neuronal firing rates and synaptic reoccupation in the deafferented medial vestibular nucleus (Lacour et al., 1991; Tighilet and Lacour, 1995; Lindner et al., 2019). Thus, EGb 761 pharmacologically targets key processes of adaptive neural plasticity following peripheral vestibular lesions (Tighilet et al., 2019; Zwergal et al., 2022).

The effect on central vestibular plasticity in animal models was primarily conveyed by the specific Ginkgo biloba terpene lactones ginkgolide A and B (Lindner et al., 2019), at plasma levels similar to those achieved through the daily intake of 240 mg of EGb 761 in humans (Woelkart et al., 2010). The results of our study are consistent with those of previous clinical studies that have demonstrated the beneficial effects of EGb 761 in peripheral and central vestibular syndromes (Hamann, 2007). A significant improvement in subjective vertigo symptoms and quality of life was observed in 70 patients with unilateral vestibulopathies after 3 months of EGb 761 treatment compared to a placebo (Haguenauer et al., 1988). Other clinical trials have demonstrated that EGb 761 increases exercise-induced postural recovery more than placebo in patients with peripheral vestibular disorders (Hamann, 1985; Orendorz-Fraczkowska et al., 2002). A randomised, placebo-controlled trial involving 40 patients with central vestibular vertigo indicated that, after a treatment period of 6 months, EGb 761 combined with vestibular exercises significantly improved both patient-rated vertigo intensity and physician-rated global functioning, compared to placebo (Heide et al., 2022).

In addition to its effects on the central nervous system, EGb 761 appears to have beneficial effects on inner ear homeostasis, as documented primarily in preclinical research on cochlear function. These include reducing inflammatory responses (Dogan et al., 2018) and hair cell apoptosis (Nevado et al., 2010; Yang et al., 2011) following drug-, age- or noise-induced cochlear damage. Similar positive therapeutic effects of EGb 761 have also been found to prevent toxic damage to vestibular hair cells in rats (Tian et al., 2013). Given the similarities in the morphogenesis of auditory and vestibular hair cells (Fritzsch and Straka, 2014), it seems plausible that EGb 761 will have neuroprotective effects on the inner ear epithelium of the vestibular system, contributing to regeneration following peripheral vestibular damage (Cassel et al., 2019).

It is important to note that the results of our clinical trial are subject to several limitations. Studies lacking randomisation and control groups are generally subject to inherent biases, which complicates the assessment of treatment effects. Information on the natural progression of vertigo and dizziness, as well as long-term outcomes, is limited because data on untreated patients is scarce. However, the course varies greatly depending on the underlying cause. For example, regression of symptoms is expected after acute unilateral vestibulopathy by mechanisms of central vestibular compensation (Lacour et al., 2016). Nevertheless, a significant proportion patients may still experience dizziness 6 months after the onset of symptoms (Van Laer et al., 2025a). Anxiety traits and fluctuations in vestibular excitability are generally considered to be risk factors for symptom chronicity in various peripheral and central vestibular disorders (Best et al., 2009; Schuhbeck et al., 2022; Van Laer et al., 2025b). Overall, for the heterogeneous patient population in our study it is difficult to estimate the rate of spontaneous improvement. Overall, it is difficult to estimate the rate of spontaneous improvement for the heterogeneous patient population in our study.

In addition to the pharmacological effects of EGb 761, non-specific effects such as lifestyle adaptation, additional balance training, and the natural progression of the disease may have influenced the outcome. For episodic vertigo/dizziness syndromes in particular, regression to the mean has often been discussed as a potential source of symptomatic improvement over time (Barnett et al., 2005). Consequently, patients with chronic vestibular disorders report a poorer quality of life compared to those with episodic vestibular disorders, even after correction for confounders (Strobl et al., 2023). Therefore, further research is needed to determine whether the better therapeutic effect of EGb 761 treatment in patients with episodic versus chronic vertigo/dizziness is specific to the pharmacological action of this drug in these disorders. However, as mentioned above, the design of this cohort study was chosen to reflect everyday clinical practice, with the aim of generating hypotheses and performing descriptive statistical analyses. Given the lack of information on how the temporal dynamics and chronicity of vertigo/dizziness affect the efficacy of EGb 761 treatment, this approach is considered reasonable. The reliability and integrity of the data are substantiated by consistent results obtained from diverse analyses of subjective and objective vertigo-related outcomes. Nevertheless, future studies are required to investigate the hypotheses generated by this study.

## 5 Conclusion

Symptomatic treatment with 240 mg of EGb 761 per day for 12 weeks in this pragmatic study was shown to be safe and well tolerated. After 6 weeks, approximately 64% of patients showed an improvement of their complaints, rising to 72% by week 12. Subgroup analyses revealed that patients with episodic vertigo/dizziness improved more than those with chronic vertigo/dizziness. Furthermore, a better response was observed in patients with a symptom duration of up to 6 months than in those with a duration of more than 6 months. However, within the setting of this pragmatic study, it is not possible to discriminate between the effects of treatment, self-healing, or the natural course of the disease.

## Data Availability

The original contributions presented in the study are included in the article/supplementary material, further inquiries can be directed to the corresponding author.
